# Concerning the Opening of Abscesses

**Published:** 1863-04

**Authors:** 


CONCERNING THE OPENING OF ABSCESSES. *By Nullopath.*—To discriminate between an abscess and certain other tumors which resemble purulent collections, requires considerable experience and skill. It also requires tact to determine when an abscess is ready for the lancet or bistoury. We judge of the contents of a tumor by the soft, doughy, fluctuating impression made upon the fingers while examining it. The history of the case furnishes additional evidence, so that we rarely fall into a faulty diagnosis. Cases may be cited, however, in which the ordinary signs are quite deceptive. For instance: two lymphatic glands near each other, appearantly forming one tumor in the neck or groin, may swell, but in the course of a few days the inflammation lessens, and a yielding and seemingly fluctuating spot is found at about the center of the swelling. Every indication favors the opinion that suppuration has commenced. It is decided that the knife must be used to evacuate the pus. In goes the bistoury, but no flow follows except blood. You feel piqued at the mistake, and the patient shows a little displeasure, perhaps hints at blundering.

Another example: A tumefied condition of the breast, ankle, or other part of the body, is presented for treatment. The history of the case is not very satisfactory, nor is the appearance of the swelling that of a common abscess, but the feeling imparted to the fingers is quite conclusive. The lancet is plunged in with confidence. No pus, but venous blood oozes from the wound. What is the trouble? Is not the incission deep enough, or in the right direction? The probe is used to explore the wound, or the lancet is made to perform a deeper, wider cut. A mistake has been made in the diagnosis. Instead of an abscess, a carcinomatous disease is encountered; and the patient's sufferings are increased, and his death hastened by this laying open of a malignant growth.

Varicose enlargements in the legs *feel* like purulent collections, but in color they are like a vein. Besides, their history is not that of an abscess.

An aneurism feels very much like a chronic abscess, yet the history of the case, the pulsation of the arterial tumor, and the peculiar aneurismal whirring, are sufficiently differential to keep us clear of a wrong diagnosis.

Pulsation alone is slender evidence of aneurism. I have noticed repeatedly that abscesses over the carotid, axillary, and femoral arteries partake of the throbbing of the vessel beneath. It is, however, a different pulsation from that of aneurism, and need not mislead. An acute abscess is painful under pressure; it presents a hard surface, except at one spot, where the walls have yielded for the purpose of spontaneous evacuation. An aneurismal tumor is not painful under pressure, and its entire surface is uniform in yielding to the pressure of the finger. If the flow of blood be impeded on the cardiac side of an aneurism, the tumor will very much diminish in size. An abscess would not be affected by interrupted circulation.

If abscesses of the pharynx be allowed to break spontaneously, the patient will be in danger of suffocation, especially if asleep. Cases of this kind are not unfrequent. To guard against accidents of this kind, pharyngeal abscesses should be opened as soon as it can be determined that pus is present. A straight, sharp-pointed bistoury, the blade being wound with a strip of adhesive plaster to within a half inch of the point, is a proper instrument to be used. Generally, the abscess forms in or about one of the tonsils, and a little care should be used, that the puncture does not reach the internal carotid artery which is not very deep.

There is not much danger of carrying a lancet or bistoury clear through both walls—the superficial and the deep—of an abscess, and into large bloodvessels beneath. The swelling of an abscess is generally outwards, or in the direction of least resistance, so that the superficial wall is removed from the vessels. Ordinarily, a puncture in the axillary space is dangerous, but in case of an abscess in that region, the swelling and accumulation of pus remove the part to be incised to quite a distance from the nerves and vessels beneath.

Much has been said and written about evacuating psoas abscesses, and others of a "cold" or scrofulous character, by means of a valvular opening, and under water, to obviate the entrance of air. I think that Abernethy was the first to advocate this process, and many eminent surgeons since have attached the highest importance to this manner of opening chronic abscesses. I have tried this and other rational plans that have been presented, yet I have no praises to bestow upon either. I am satisfied that nothing is gained by attempting to exclude the air from these large pyogenic cavities. They are slow to heal, and if the air does not enter when the puncture is first made, it will before the healing process commences, therefore nothing is gained. It is better, at first, to open the abscess freely, and then, if the cavity be easily reached, let it be injected with a very weak solution of the chloride of zinc. Long crooked sinuses leading from the inside of the thigh, for instance, to the lumbar region, can not be injected with any hope of benefit.

Abscesses near the anus should be opened early, and the cavity washed daily with the zinc solution, to prevent the formation of a fistula.

A mature whitlow is an abscess, and should be freely opened; but it is bad surgery to follow the directions of those who advocate, before suppuration has commenced, the opening to the bone of every progressive fellon The true practice is, to suppress the forming whitlow, by keeping it constanly under the influence of powerful anodynes, topically applied. If the disease be not presented for treatment until suppuration has commenced, it is then best to poultice the finger for a day or two, and then evacuate the pus by an incision.—*Journal of Rational Medicine.*

